# The Binding Affinity of Small Molecules with Yam Tyrosinase (Catechol Oxidase): A Biophysical Study

**DOI:** 10.1155/2019/8284968

**Published:** 2019-10-10

**Authors:** Tabassum Mulla, Sushama Patil, Srinivas Sistla, Jyoti Jadhav

**Affiliations:** ^1^Department of Biotechnology, Shivaji University, Kolhapur 416004, India; ^2^Institute for Structural Biology, Drug Discovery and Development, Virginia Commonwealth University, Richmond, VA 23298, USA

## Abstract

Yam tyrosinase has become an economically essential enzyme due to its ease of purification and abundant availability of yam tubers. However, an efficient biochemical and biophysical characterization of yam tyrosinase has not been reported. In the present study, the interaction of yam (*Amorphophallus paeoniifolius*) tyrosinase was studied with molecules such as crocin (*Crocus sativus*), hydroquinone, and kojic acid. Surface plasmon resonance (SPR), fluorescence spectroscopy, and circular dichroism techniques were employed to determine the binding affinities and the changes in secondary and tertiary structures of yam tyrosinase in the presence of four relevant small molecules. Hydroquinone and crocin exhibited very low binding affinities of 0.24 M and 0.0017 M. Due to their apparent weak interactions, competition experiments were used to determine more precisely the binding affinities. Structure-function interrelationships can be correlated in great detail by this study, and the results can be compared with other available tyrosinases.

## 1. Introduction

Tyrosinase is a universal enzyme present in all living organisms. It is involved in the conversion of monophenols to diphenols by monophenolase activity and further conversion of diphenols into quinones by diphenolase activity. Tyrosinase and two other accessory enzymes called the tyrosinase-related proteins (Trp1 and Trp2) are mainly responsible for the synthesis of melanin in mammals. The excess synthesis of melanin may lead to skin problems like melasma, freckles, lentigo senilis, and other forms of melanin hyperpigmentation [1]. Unlike animals, the tyrosinases of plants are involved in the synthesis of phenolic polymers like lignin, flavonoids, and tannins and are involved in some metabolic processes such as cellular respiration, regulation of oxidation-reduction potential, host defense, and wound healing [2, 3]. The lack of tyrosine hydroxylation is a distinct feature of plant tyrosinase compared to animal tyrosinases. Tyrosinases and catecholamines (neurotransmitters synthesized by hydroxylation of tyrosine by the action of tyrosine hydroxylase) differ in a single amino acid, G241. Understanding these differences between plant and animal enzymes will help designing effective modulators. Tyrosinase leads to undesirable browning during the processing of fruits and vegetables and thus reduces their commercial value. Therefore, tyrosinase inhibitors have been under study for some time, and the use of these inhibitors has increased in the cosmetic, medicinal, and food industries [4, 5]. Many tyrosinase inhibitors, including natural as well as synthetic inhibitors like flavonoids, polyphenols, oxyresveratrol, hydroxystilbene compounds [6], quercetin [5], triolein, and trilinolein [7], have been studied. Notably, elephant foot yam tyrosinase has been purified and biochemically characterized for baking applications. As it is abundantly available and very thermostable and has an economical method of purification, yam tyrosinase has attracted considerable attention in the food and baking industries [8].

As tyrosinase is a commercially important enzyme, its inhibition will be of interest in the cosmetics and food industries. While many different biophysical methods can be employed for enzyme inhibitor binding interaction studies, surface plasmon resonance (SPR) is a particularly robust and well-utilized method [9–12]. Recently, Patil et al. [13] used the SPR system for studying the binding events between mushroom tyrosinase and small molecules. SPR is a biosensor-based binding technique used for biomolecular interaction analysis. It measures the binding events between molecules at the metal surface by detecting changes in the local refractive index [14]. SPR has the features of real-time measurement, label-free interactions, and surface-sensitive response [9]. SPR has a wide range of applications in kinetic measurements (*k*_a_, *k*_d_), in binding site analysis and concentration determination, in screening of drug molecules against enzymes, and in specificity analysis.

The purpose of this study is to understand the binding of different analytes with tyrosinase enzyme (ligand). The goal was to determine the capability of inhibitors (individually or in combination) with respect to modulation of yam tyrosinase. A competitive analyte kinetics model approach was conducted to study the interactions between inhibitors like kojic acid, hydroquinone, and crocin towards yam tyrosinase. The strategy of using a combination of (inhibitors) kojic acid, hydroquinone, and crocin along with its substrate, L-DOPA was to study the binding and synergistic effect of these molecules with immobilized yam tyrosinase. Furthermore, the effect of small molecules on yam tyrosinase was determined with fluorescence spectroscopy and circular dichroism (CD) spectroscopy techniques.

## 2. Materials and Methods

### 2.1. Chemicals and Reagents

Sensor chip series S CM5, N-ethyl-N'-(dimethylaminopropyl)-carbodiimide (EDC), N-hydroxysuccinimide (NHS), and ethanolamine HCl, as well as sampling vials, were obtained from GE Healthcare Life Sciences, Uppsala, Sweden. The hydroquinone and L-3 dihydroxyphenylalanine (L-DOPA) compounds were obtained from HiMedia, Mumbai, India. Kojic acid and crocin were obtained from Sigma-Aldrich, Mumbai, India. All other chemicals were of the highest purity and analytical grade. Milli-Q (millipore) water was used for preparing buffers and reagents.

### 2.2. Enzyme Source

The yam tyrosinase was extracted from *Amorphophallus paeoniifolius* tubers and partially purified by passing it through the ultracentrifugal cutoff filter units. This extract was then subjected to a DEAE cellulose column for purification and confirmed by SDS-PAGE. This purified yam tyrosinase was used for further study as described in the previous report [15].

### 2.3. Enzyme Activity Assay

#### 2.3.1. Diphenolase Activity Assay

The diphenolase activity of yam tyrosinase was performed by measuring the dopachrome accumulation at 475 nm (Ɛ_dopachrome_ = 3400 M^−1^ cm^−1^) by using L-DOPA as a substrate with little modifications to published procedures [16]. The reaction mixture (3 mL) contained 2 mM of L-DOPA in 50 mM potassium phosphate buffer (pH 7.0); a portion of 100 *μ*L of the enzyme was used for the activity assay. The reaction was carried out at a constant temperature of 30°C using a Shimadzu UV visible spectrophotometer. IC_50_ values were calculated as per the formula reported earlier [13].

### 2.4. Surface Plasmon Resonance (SPR) Studies

SPR interaction studies were carried out using a Biocore X100 optical biosensor (GE Healthcare Life Sciences, Uppsala, Sweden). All the SPR measurements were performed in phosphate buffer saline (PBS; 10.1 mM Na_2_PO_4_, 1.8 mM KH_2_PO_4_, 137 mM NaCl, 2.7 mM KCl, pH 7.4, 0.005% P20). Initially, working dilutions were prepared from the analyte stock solution in PBS only and then allowed to flow through the sensor surface. Biocore control software version 2.2 was used for data collection. Refractive index changes as a function of time under constant flow condition are monitored by the system, and accordingly, experiments were performed. The net increase in refractive index over time compared with that of buffer alone gives the relative amount of inhibitor bound to the tyrosinase. There is an inline subtraction of reference surface during the run. This change is usually reported in terms of response units (RUs). The surface of the chip is washed with PBS (running buffer) between each concentration [13].

#### 2.4.1. Enzyme Immobilization

The method of amine coupling was employed for the immobilization of yam tyrosinase which was dissolved (50 *μ*g/mL) in 0.1 M sodium acetate buffer with pH value 4.5 as per the pH scouting study in [15]. Yam tyrosinase was immobilized on sensor chip series S CM5. Flow cell on the chip surface was activated for 7 min using a 1 : 1 mixture of 100 mM N-ethyl-N-9 dimethyl amino propyl : carbodiimide (EDC) and 100 mM N-hydroxysuccinimide (NHS) (both dissolved in water), and subsequently, tyrosinase was injected for 7 min, followed by injection of 1 M ethanolamine, pH 8.5 to block nonreactive sites. For this study, flow cell 3 was blank immobilized (without protein) as reference.

#### 2.4.2. Competitive Analyte Study Using SPR

To study the inhibition of yam tyrosinase, a few potent inhibitors were selected. The compounds kojic acid, hydroquinone, and crocin, which are known potent tyrosinase inhibitors, were used to study their comparative and synergistic effects on yam tyrosinase. Crocin and kojic acid are natural tyrosinase inhibitors, whereas hydroquinone is a synthetic inhibitor. The grouping of inhibitors was simply based on the efficiency, size, solubility, and origin of inhibitors. Initially, the inhibitors kojic acid, hydroquinone, crocin, and substrate L-DOPA were dissolved in 10 mM PBS containing 0.005% P20 and were injected over a sensor chip to study the interactions between inhibitors and yam tyrosinase. PBS was used as the running buffer. The flow rate was constant (45 *μ*L/min) throughout the experiment. The contact time (association time) and dissociation time were fixed to 120 s. Experiments were performed with different concentrations of individual inhibitors based on their solubilities: crocin (0.156–2.5 mM), hydroquinone (0.625–10 mM), kojic acid (0.0156–0.125 mM), and L-DOPA (5–80 mM). These concentrations were optimized by repeating the SPR experiments several times to achieve appropriate binding interactions. The combination of small molecules was prepared as follows for the competition experiments: hydroquinone + crocin, kojic acid + hydroquinone, and crocin + L-DOPA, with the same concentrations as above mixed in 1 : 1 molar proportions and then allowed to flow over the sensor chip surface. Regeneration was carried out with 10 mM glycine pH 2.5 for 30 s. Binding analysis was evaluated using the heterogeneous analyte kinetics model. After each cycle of regeneration, the activity of the enzyme was checked by injecting a single concentration of L-DOPA, and RU response was observed [13]. The data analyses were done with Biacore X100 evaluation software version 2.2, and data were fit to two-state binding and/or heterogeneous analyte binding.

### 2.5. Fluorescence Spectroscopy Study

The fluorescence intensities were recorded using an MY14410002 fluorescence spectrophotometer (Agilent Technologies, USA) with an excitation wavelength (*λ*_ex_) of 280 nm and a slit width of 5 nm. To determine protein fluorescence, yam tyrosinase (2 mg/mL) was prepared in phosphate buffer [10 mM, pH 7.0]. The maximum emission wavelength (*λ*_em_) for tyrosinase was 307 nm. The effect of the inhibitors kojic acid, hydroquinone, and crocin individually and in combination was studied on yam tyrosinase by fluorescence spectroscopy. Inhibitors were prepared in 50 mM potassium phosphate buffer with concentrations of crocin (1 mM), hydroquinone (1 mM), and kojic acid (0.1 mM) in 3 mL of reaction mixture including 0.3 mL of tyrosinase. At the same time, cocktails were prepared with same concentrations as mentioned above to study their combinational effect on tyrosinase. The enzyme along with buffer was used as a control. The change in the spectra obtained after addition of inhibitors to yam tyrosinase was observed and recorded as an intensity vs. wavelength plot [17].

### 2.6. Circular Dichroism (CD) Spectroscopy

The CD spectra were recorded on a Jasco J-1500 CD-spectropolarimeter to determine the change in the content of the secondary structure of yam tyrosinase in the presence of inhibitors. The protein sample of 0.2 mg/mL was prepared in 10 mM potassium phosphate buffer, pH 7.0. Scanning was performed at 25°C using a 2 mm path length quartz cuvette with 1 s differential integration time at a scan rate of 100 nm/min. The inhibitors like crocin (1 mM), hydroquinone (1 mM), kojic acid (0.1 mM), and substrate L-DOPA at a concentration of 1 mM were prepared in the same phosphate buffer. The combinations of small molecules were also prepared with the same concentrations. The protein solution was incubated with crocin, hydroquinone, kojic acid, L-DOPA, and different mixtures for 10 min, and then the spectra were recorded. The enzyme along with the buffer was used as a control. All spectra were collected in triplicate from 190 to 250 nm, and the background was corrected against buffer blank. The results were calculated in terms of molar ellipticity (*θ*) (deg cm^2^/dmol) [18]. Reed's reference (database) was used for the analysis of circular dichroism data [19].

## 3. Results and Discussion

Tyrosinase has become a vital drug target and potent inhibitor for it will have profound use in the cosmetics, food, and pharmaceutical industries [17, 20]. There are huge differences between plant, fungal, animal, and human tyrosinases, and effective inhibitors are needed for tyrosinases individually. Despite many reports on tyrosinases, a thorough characterization is not available, and high-affinity inhibitors are yet to be identified [21]. Many of the chemically synthesized tyrosinase inhibitors such as hydroquinone, kojic acid, arbutin, ascorbic acid, oxyresveratrol, hydroxystilbene, ellagic acid, and gallic acid are already in use as skin whitening agents in cosmetics with few drawbacks [22–24]. Recently, thiopurine drugs such as thioguanine were repurposed as tyrosinase inhibitors [25]. In addition, natural tyrosinase inhibitors, e.g., 1-(2,4-dihydroxyphenyl)-3-(2,4-dimethoxy-3-methylphenyl)propane found in the medicinal plant *Dianella ensifolia* [26] are also under study. Also, extracts of *Scutellaria* species have displayed weak tyrosinase inhibition [27]. Since ancient times, antityrosinase activities were displayed by the stem bark powder of *Hesperethusa crenulata*, *Naringi crenulata,* and *Limonia acidissima* and are traditionally used in skin whitening treatment in the Indian subcontinent and Southeast Asia [28]. Similarly, the extracts obtained from leaves and stems of *Podocarpus elongatus*, *P. falcatus*, *P. henkelii*, and *P. latifolius* showed tyrosinase inhibition and have also been used in traditional medicine in Southern Africa [2].

As discussed earlier, SPR spectroscopy can evaluate the binding interactions between molecules. In this study, SPR techniques were employed for studying the binding affinities and kinetics of yam tyrosinase against inhibitor molecules. As surface plasmon resonance is a biosensor-based technique, it can be rigorously used in drug discovery and presents significant advantages including fast response times and the ability to detect multianalytes simultaneously. SPR is extensively used in biochemistry and bioanalytical chemistry to characterize the interactions between biological molecules, e.g., in antigen-antibody interactions and RNA-DNA hybridizations, in the diagnosis of bacteria, virus-induced diseases, biosimilarity, serum quantification, and investigation of hormones, steroids, immunoglobulins, blood coagulation factors, and so on [29].

**Figure 1:**
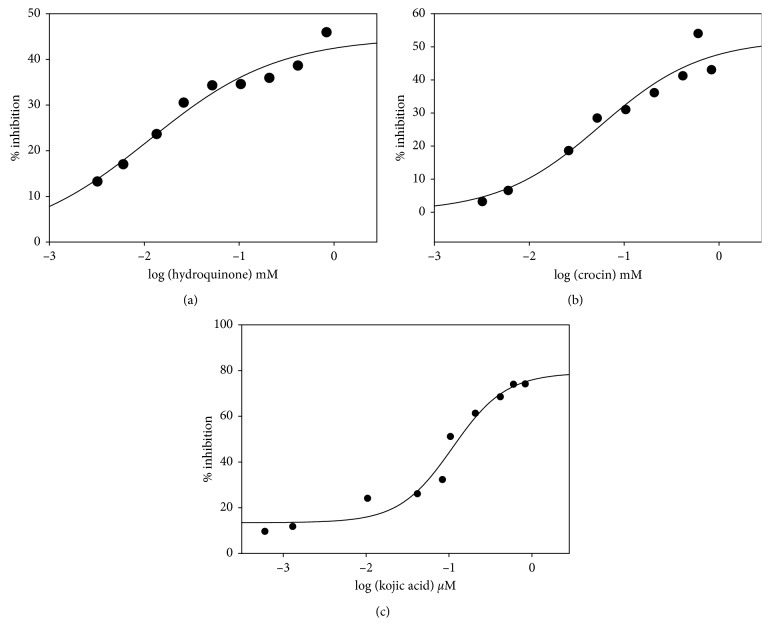
Effect of compounds hydroquinone (a); crocin (b); and kojic acid (c) on the diphenolase activity of yam tyrosinase using L-DOPA as the substrate.

In the present study, yam tyrosinase was immobilized on gold sensor chip (CM5) dextran matrix using an amine coupling method with the final response of 1300 units (RUs) [15]. Small molecules were passed over the yam tyrosinase immobilized on the chip surface, and the association (*k*_a_) and dissociation rates (*k*_d_) of all specific inhibitors were calculated (Table 1). A slower binding phase was represented by *k*_a1_ and *k*_d1,_ while the rapidly changing transition phase is resembled by *k*_a2_ and *k*_d2_. Kojic acid shows a *K*_D_ of 6.39 × 10^−6^ M and therefore has the highest affinity towards yam tyrosinase, e.g., compared to crocin having *K*_D_ of 0.001662 M followed by hydroquinone with *K*_D_ = 0.2432 M. L-DOPA, used as substrate, showed maximum affinity with *K*_D_ = 1.35 × 10^−6^ M as per an earlier report [15]. The IC_50_ values and potency experiments were calculated for the inhibitors: hydroquinone (1.5 mM), crocin (1 mM), and kojic acid (0.1 *μ*M) (Figures 1(a)–1(c)).

**Figure 2:**
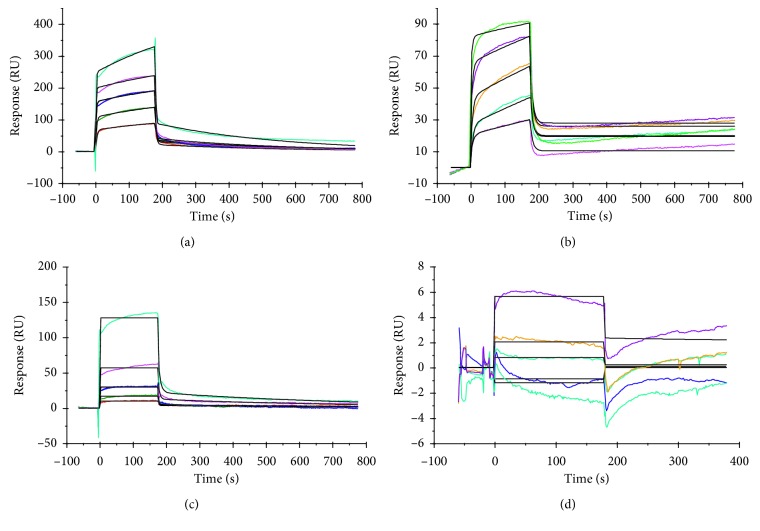
Binding sensogram for compounds having interactions with immobilized yam (*Amorphophallus paeoniifolius*) tyrosinase. Flow rate was maintained at 45 *μ*L/min. Contact time and dissociation time were kept at 120 s and 200 s. Regeneration was carried out using 10 mM glycine HCl at pH 2.5 for 30 s and at 30 *μ*L/min. The data analysis was performed with Biacore X100 evaluation software version 2.0.1, and data were fit to two states. Each concentration represents sensorgram in the increasing order of curves. Crocin with concentrations (0.156, 0.312, 0.625, 1.25, and 2.5 mM) was injected over the enzyme surface (a). Kojic acid with concentrations (0.0156, 0.0312, 0.0625, 0.125, and 0.25 mM) was injected over the enzyme surface (b). Hydroquinone with concentrations (0.625, 1.25, 2.5, 5.0, and 10 mM) was injected over enzyme surface (c). L-DOPA with concentrations (5, 10, 20, 40 and 80 mM) was injected over the enzyme surface (d).

**Figure 3:**
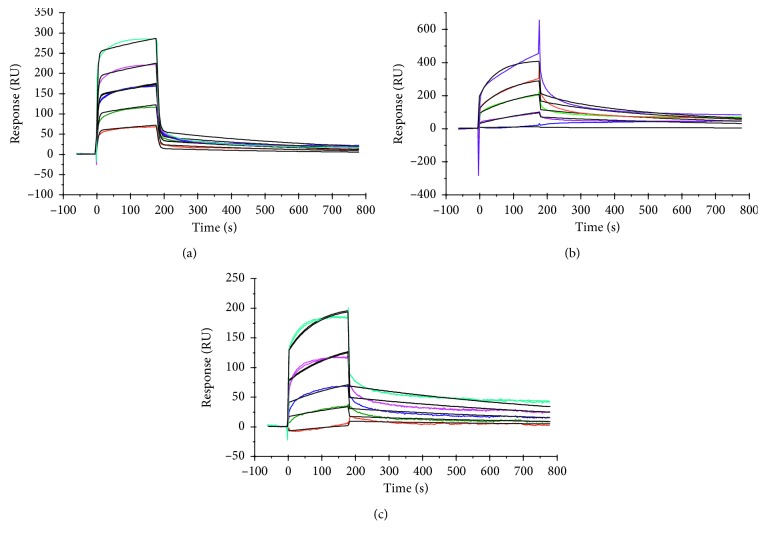
Binding sensogram for cocktail interactions with immobilized yam (*Amorphophallus paeoniifolius*) tyrosinase. Flow rate was maintained at 45 *μ*L/min. Contact time and dissociation time were kept at 120 s and 200 s. Regeneration was carried out using 10 mM glycine HCl at pH 2.5 for 30 s and at 30 *μ*L/min. The data analysis was performed with Biacore X100 evaluation software version 2.0.1, and data were fit to a heterogenous analyte model. Each concentration represents sensorgram in the increasing order of curves. Concentrations of hydroquinone (0.625, 1.25, 2.5, 5.0, and 10 mM) + crocin (0.156, 0.312, 0.625, 1.25, and 2.5 mM) were injected over the enzyme surface (a). Concentrations of kojic acid (0.0156, 0.0312, 0.0625, 0.125, and 0.25 mM) + hydroquinone (0.625, 1.25, 2.5, 5.0, and 10 mM) were injected over the enzyme surface (b). Concentrations of crocin (0.156, 0.312, 0.625, 1.25, and 2.5 mM) + L-DOPA (5, 10, 20, 40, and 80 mM) were injected over the enzyme surface (c).

Competition of analytes was also studied and the data were fit to a heterogeneous analyte kinetic model. The molecules were small, with molecular weights for hydroquinone (110.11 g/mol), kojic acid (142.11 g/mol), and L-DOPA (197.19 g/mol), except for crocin (976.972 g/mol). It is clear that inhibitors like crocin with high molecular weight give higher responses (Figure 2(a)) than kojic acid (Figure 2(b)) and hydroquinone (Figure 2(c)) and even for the L-DOPA substrate (Figure 2(d)). Competition experiments help resolve this effect by mixing both low- and high-molecular-weight inhibitors and performing heterogeneous analyte kinetics in which the combination of molecules was allowed to run over the sensor chip surface [30]. The competing analytes hydroquinone + crocin (Figure 3(a)), kojic acid + hydroquinone (Figure 3(b)), and crocin + L-DOPA (Figure 3(c)) showed an increase in responses compared with their individual molecules. In addition, *K*_D_ values decreased for competing molecules: 2.14 × 10^−7^ M and 7.15 × 10^−7^ M for hydroquinone + crocin, 5.31 × 10^−7^ M and 2.26 × 10^−6^ M for kojic acid + hydroquinone, and 2.4 × 10^−7^ M and 2.3 × 10^−7^ M for crocin + L-DOPA (Table 2). The biosensor response of heterogeneous analytes depends upon association and dissociation rates (*k*_a_, *k*_d_) of both high- and low-molecular-weight inhibitors [13]. The data derived from this experiment also showed that the combination of inhibitors (natural source + synthetic source) was more effective than a combination of two synthetic inhibitors. Hence, such a combination of inhibitors can be used to inhibit the enzyme more efficiently.

Therefore, the data derived from the experiment gives two different *K*_D_ values, *K*_D1_ for the first inhibitor and *K*_D2_ for the second inhibitor in a combination. The observed *K*_D_ values from both the tables (Tables 1 and 2) ensure that binding affinities of crocin, hydroquinone, kojic acid, and L-DOPA in combination had increased affinity or kinetic rates than that of an individual molecule. As tyrosinase is an enzyme with dual activity, it thus possesses two domains. Therefore, both the inhibitors in a mixture compete and bind either of the domains of yam tyrosinase immobilized on a sensor chip and give different *K*_D_ values.

**Figure 4:**
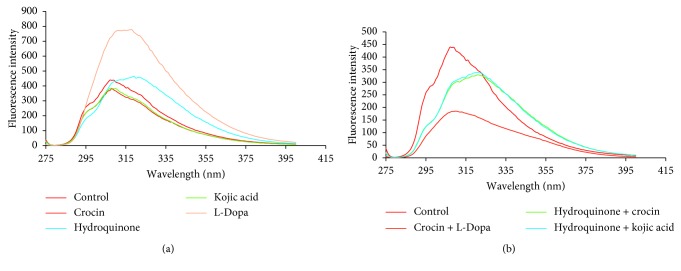
Fluorescence emission spectra of yam tyrosinase at (*λ*_ex_) 270 nm showing change in conformation in presence of inhibitors, kojic acid, hydroquinone, and crocin (a) and in mixture of inhibitors such as hydroquinone + crocin, kojic acid + hydroquinone, and crocin + L-DOPA (b).

These kinetics results were also supported by fluorescence spectroscopy studies. Fluorescence intensities of yam tyrosinase were recorded before and after addition of the crocin, hydroquinone, and kojic acid inhibitors, and conformational alteration in yam tyrosinase due to the inhibitors was confirmed (Figure 4(a)). Tryptophan fluorescence has been frequently examined among the intrinsic aromatic fluorophores in tyrosinase molecules so as to observe conformational changes. Yam tyrosinase has strong fluorescence emission with a peak at 307 nm upon excitation (*λ*_ex_) at 270 nm. There is no spectral shift in the presence of crocin and kojic acid with fluorescence emission peaks, while hydroquinone showed a red shift (bathochromic shift) in the wavelength with an emission peak at 319 nm.

Also, heterogeneous mixtures of inhibitors and substrate also proved dramatic tertiary structural changes. The fluorescence emission spectra of yam tyrosinase were collected for combinations of molecules that exhibited inhibition of tyrosinase by altering its polarity (Figure 4(b)). Combinations of hydroquinone + crocin and kojic acid + hydroquinone both exhibit red shifts with concomitant decreases in fluorescence intensities at the maximum emission wavelength of 321 nm. In contrast, crocin + L-DOPA showed a change in fluorescence intensity at a maximum emission of 310 nm. The data obtained from these fluorescence studies also supported the competition experiments. A few reports on mushroom tyrosinase have quoted the change in fluorescence before and after the addition of inhibitors such as flavonoids [17], glycolic acid [31], and dihydromyricetin [32]. The conformational change was evaluated by measuring the fluorescence intensity of the mushroom tyrosinase after addition of condensed tannins from *Ficus virens* showing antityrosinase activity [33]. Measurement of change in fluorescence showed that there is a phthalic acid-induced change in the active site of mushroom tyrosinase by indirect binding [34].

**Figure 5:**
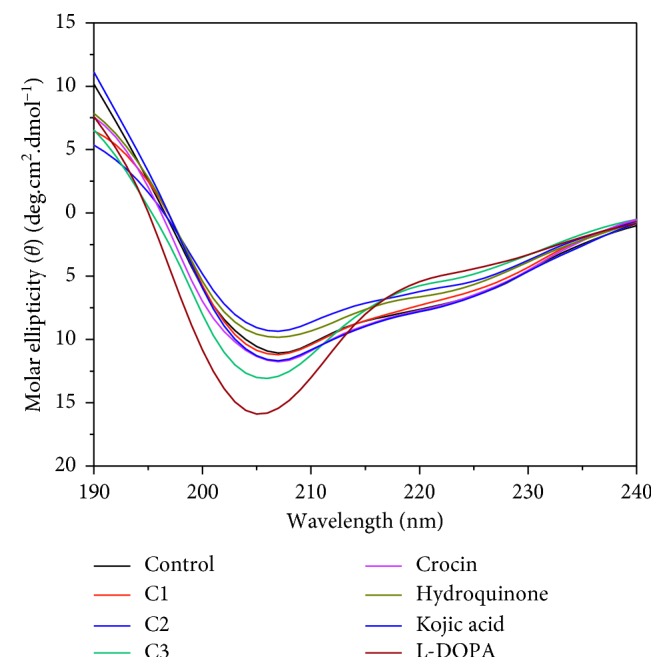
CD spectrum of yam tyrosinase as a control (black curve) and control with cocktail No. 1 (red curve), cocktail No. 2 (dark blue curve), cocktail No. 3 (blue-green curve), crocin (dark pink curve), hydroquinone (yellow-green curve), kojic acid (violet curve), and L-DOPA (brown curve) with 1 mM concentration each. C1: hydroquinone + crocin, C2: kojic acid + hydroquinone, and C3: crocin + L-DOPA.

The structural changes in yam tyrosinase in the presence of inhibitors were also confirmed by circular dichroism spectroscopy (Figure 5). Karbassi et al. [18] performed CD spectroscopy studies to determine the effect of SDS on mushroom tyrosinase. Later, Patil et al. [35] showed a change in conformation of mushroom tyrosinase in the presence of tannic acid, pyrogallol, catechol, saffron, and phloroglucinol inhibitors. In this work, we examined the effect of our set of inhibitors and their heterogeneous mixtures on yam tyrosinase. All inhibitors, substrates, and combinations showed changes in secondary structures of the enzyme. The mixture of inhibitors showed a more significant change in the secondary structure of the native enzyme than individual inhibitors. The percent changes in secondary structures of yam tyrosinase in the presence of individual inhibitors and mixtures of inhibitors are given in Table 3. In summary, combinations of different inhibitors were more efficient in changing the conformation of the protein than individual inhibitors. The CD spectroscopy study thus confirmed the SPR and fluorescence spectroscopy studies in which combinations of inhibitors proved to be better inhibitors than just individuals.

The present work presents a heterogenous analytical study carried out to determine the interactions of two molecules simultaneously towards an enzyme that results in two sets of rate constants for each one. Response contributions from both analytes are taken into account by considering molecular weight and concentration of the analytes. In cases of heterogenous analytes, molecules binding to both sites of the target molecule are not released from the surface until dissociation occurs at both sites; so the observed dissociation rate is much slower compared to a single site binding with higher affinity. This concept is known as avidity. This study also revealed that low-molecular-weight molecules give low response, whereas a high-molecular-weight molecule showed considerably higher response in the Biacore studies. Similarly, fluorescence and circular dichroism spectroscopic studies displayed the efficiency of active compound mixtures in inhibiting tyrosinase.

## 4. Conclusion

The present study showcased the effect of combination of natural and synthetic inhibitor molecules along with substrate. The mixtures of inhibitors proved to inhibit yam tyrosinase more efficiently than individual molecules. A combination of inhibitors has a very significant effect on the kinetics and secondary and tertiary structures of yam tyrosinase. A different approach to study the heterogeneous analyte kinetics model was employed to derive *K*_D_ values for competing molecules. *K*_D_ values in the mixture have higher affinity than those of the individual inhibitors, with the net result being in increased binding affinity for both. These kinds of data will be useful in designing synergistic inhibitors in a similar enzyme kinetics study. Such an enzyme inhibition approach should be encouraged for effective inhibition of enzyme. This method can provide both qualitative and quantitative data on binding kinetics. The significance of the present work is that all the experiments were performed on a single immobilized enzyme surface making results more reliable and repeatable. The sensors with such strategies can be used in drug discovery and development process.

## Figures and Tables

**Table 1:** Interaction kinetics of yam tyrosinase with different inhibitors fit to a two-state model using Biacore evaluation software.

Compounds	*k*_a1_ (1/ms)	*k*_d1_ (1/s)	*k*_a2_ (1/s)	*k*_d2_ (1/s)	*K*_D_ (M)
Kojic acid	334.3 ± 16	0.03182 ± 0.0013	5.39 × 10^−3^ ± 7.8 × 10^−5^	0.03877 × 10^−3^ ± 6.5 × 10^−4^	6.39 × 10^−6^
Hydroquinone	2.893 ± 0.13	0.9166 ± 0.02	1.569 × 10^−3^ ± 3.5 × 10^−5^	5.185 × 10^−3^ ± 1.3 × 10^−4^	0.2432
Crocin	224.3 ± 11	0.5772 ± 0.025	3.525 × 10^−3^ ± 9.7 × 10^−5^	6.429 × 10^−3^ ± 1.4 × 10^−4^	0.001662
L-DOPA	9.54 × 10^4^	0.5089 ± 0.085	0.001544 ± 1.50 × 10^4^	5.23 × 10^−4^ ± 8.40 × 10^−5^	1.35 × 10^−6^

**Table 2:** Interaction kinetics of yam tyrosinase with a mixture of two different inhibitors fit to a heterogeneous analyte model.

Compounds	*k*_a1_ (1/ms)	*k*_d1_ (1/s)	*k*_a2_ (1/s)	*k*_d2_ (1/s)	*K*_D1_ (M)	*K*_D2_ (M)
Hydroquinone + crocin	32.99 × 10^4^ ± 3.2 × 10^4^	0.0705 ± 2.4 × 10^−3^	5.93 × 10^4^ ± 7.80 × 10^3^	4.24 × 10^−2^	2.14 × 10^−7^	7.15 × 10^−7^
Kojic acid + hydroquinone	6.36 × 10^4^ ± 26 × 10^3^	0.03372 ± 2.3 × 10^−3^	6.7 × 10^4^ ± 2.0 × 10^4^	1.51 × 10^−1^	5.31 × 10^−7^	2.26 × 10^−6^
Crocin + L-DOPA	6.62 × 10^3^ ± 7.6 × 10^3^	0.001587 ± 0.3 × 10^−3^	11.05 × 10^4^ ± 1.2 × 10^5^	2.54 × 10^−2^	2.4 × 10^−7^	2.3 × 10^−7^

*K*_D1_ and *K*_D2_ represent rate constants of first and second inhibitors in a cocktail, respectively.

**Table 3:** Percent changes in secondary structures of yam tyrosinase in the presence of inhibitors.

% change	Compounds							
	Control	Crocin	Hydroquinone	Kojic acid	L-DOPA	C1	C2	C3
*α*-helix	15.6	9.5	11.9	14.9	8.2	7.2	7.9	7.1
*β*-sheet	33.8	36.5	35.2	32.3	25.8	39	40.2	30.9
Β-turns	2.1	4.6	4.5	4.5	13.5	5.2	3.2	11

C1: hydroquinone + crocin; C2: kojic acid + hydroquinone; and C3: crocin + L-DOPA. Data presented as the mean of three replicates with standard deviations (*n* = 3).

## Data Availability

The figures, tables, and assay conditions used in preparing the results and conclusions to support the findings of this study are included within the article.
